# Understanding the association of *Escherichia coli* with diverse macroalgae in the lagoon of Venice

**DOI:** 10.1038/srep10969

**Published:** 2015-06-04

**Authors:** Grazia M. Quero, Luca Fasolato, Carla Vignaroli, Gian Marco Luna

**Affiliations:** 1 Institute of Marine Sciences (CNR – ISMAR), National Research Council, Venezia, Italy; 2 Department of Comparative Biomedicine and Food Science, University of Padova, Italy; 3 Department of Life and Environmental Sciences, Polytechnic University of Marche, Ancona, Italy

## Abstract

Recent studies provided evidence that the macroalga *Cladopohora* in lakes hosts associated *Escherichia coli*, with consequences on the environmental and human health. We expanded these investigations to other macroalgae (*Ulva* spp., *Sargassum muticum* and *Undaria pinnatifida*) widespread in the lagoon of Venice (Italy). Attached *E. coli* were abundant, accounting up to 3,250 CFU gram^−1^ of alga. Macroalgal-associated isolates belonged to all *E. coli* phylogroups, including pathogenic ones, and to *Escherichia* cryptic clades. Attached *E. coli* showed potential to grow even at *in situ* temperature on macroalgal extracts as only source of carbon and nutrients, and ability to produce biofilm *in vitro*. The genotypic diversity of the attached isolates was high, with significant differences between algae and the overlying water. Our evidences suggest that attached populations consist of both resident and transient strains, likely resulting from the heterogeneous input of fecal bacteria from the city. We report that cosmopolitan and invasive macroalgae may serve as source of *E. coli*, including pathogenic genotypes, and that this habitat can potentially support their growth. Considering the global diffusion of the macroalgae here studied, this phenomenon is likely occurring in other coastal cities worldwide and deserves further investigations from either the sanitary and ecological perspectives.

The fecal indicator bacterium (FIB) *Escherichia coli* can persist in a variety of secondary habitats, such as water, sand, sediments and others^1^,^2^,^3^,^4^,^5^. These studies have suggested that some strains can replicate in the environment, and be reintroduced to the primary host through water and food^6^. Strains isolated from the environment display different characters than commensal strains from animals or humans^7^,^8^,^9^. In this perspective, there is a human health concern due to potential acquisition of new properties (virulence factors or antibiotic resistances)^10^ suggesting the need to study the ecology of *E. coli* in secondary habitats.

*E. coli* strains can be assigned to 8 phylogroups, seven (A, B1, B2, C, D, E and F) belonging to *E. coli sensu stricto*^11^ and one corresponding to the cryptic *Escherichia* clade I^11^, which is phylogenetically the closest lineage of *E. coli* among the five cryptic clades^12^. The cryptic lineages (II to V) have been identified in the *Escherichia* genus, which are genetically but not phenotypically distinct from *E. coli*^12^,^13^. Strains belonging to different phylogroups can occupy separate ecological niches and display diverse properties or ability to cause infections^11^,^14^. Many commensal strains belong to groups A and B1, whereas *E. coli* associated with human extraintestinal infections frequently belong to groups B2 and D^15^,^16^,^17^. Phylogroup A and B1 are typically over-represented in waters^2^,^18^ and B1 strains are more persistent in soil^19^. The primary niche of the clades III, IV and V may be outside of the host gut, and they are believed to represent environmentally-adapted *Escherichia* isolates^13^,^20^. Cryptic lineages have been suggested to unlikely represent a significant human risk^12^,^20^, but other authors reported that clade V strains isolated from marine sediments show gene repertoires and adhesion properties similar to those of pathogenic strains, suggesting potential virulence^9^. The existence of “naturalized” populations^21^, whose distribution in the aquatic environment is understudied, poses questions about the reliability of *E. coli* as a fecal indicator^12^.

Marine algae are colonized by abundant microbes^22^, with a role in the development, defence and metabolism of the plant^23^. Algal exudates are a nutrient source and contain substrates able to sustain bacterial growth^24^, such as polysaccharides and glycoproteins^25^, and algae can provide survival advantages to associated pathogenic microbes^26^. Studies in lakes showed that *Cladophora* and wracks can harbour FIB^24^,^27^,^28^,^29^,^30^. Macroalgae are a potentially favourable environment for FIB, by providing sites for adhesion, protection against UV radiations and predation, and nutrients. However, information available is limited to the freshwater macroalga *Cladophora*, while studies have not addressed widely spread macroalgal genera, such as the cosmopolitan *Ulva*^31^ or the invasive *Undaria* and *Sargassum*^32^,^33^.

The Venice lagoon is the largest in Italy (ca. 550 km^2^) and one of the largest in the Mediterranean Sea^34^. It is a highly–polluted environment, especially in the area surrounding the city, due to a variety of industrial and agricultural waste, and the presence of domestic wastes deriving from the lack of adequate sewage treatment infrastructures^35^,^36^,^37^. The lagoon has experienced several macroalgal blooms in response to eutrophication^34^,^38^. About 300 macroalgal species have been recorded in the area, among which Chlorophyceae, Rhodophyceae, Phaeophyceae and Chrysophyceae^39^, and there are continuous records of newly–introduced species. The invasive species *Sargassum muticum* (Yendo) Fensholt and *Undaria pinnatifida* (Harvey) Suringar, introduced into the lagoon in the early 1990s, are the most abundant invaders colonizing the hard substrata of the historical centre of Venice^37^. *U. pinnatifida* is a cold-temperate species originating from Asia, while *S. muticum* is a temperate species distributed along a large latitudinal range^37^. Conversely, *Ulva* species have a nearly ubiquitous distribution in a wide range of environments^40^. *Ulva* spp., *S. muticum* and *U. pinnatifida* colonize the hard substrates of the lagoon and, especially during spring, the edges of the city canals are largely covered by the algal thalli. *S. muticum* and *U. pinnatifida* reach high densities (up to 15 kg f wt m^−2^ in April–May) and considerable length (up to 7 meters)^34,41^, creating concern and problems to navigation. Investigating whether they can accumulate FIB appears important, considering the potential evolution of pathogenic strains on their surface, and the spread of contaminated thalli in areas of the lagoon hosting aquaculture farms^34^ or in bathing beaches located immediately outside the lagoon inlets.

We studied the association between *E. coli* and the dominant macroalgae in the city of Venice (Fig. 1), with the aim of i) quantifying the abundance of *E. coli* attached to the macroalgae, to test the hypothesis that seaweeds are sources or reservoirs for *E. coli*; ii) describing the population structure, by assigning isolates to the phylogenetic group or the cryptic clades; iii) performing experiments to investigate whether macroalgae support the growth of enteric bacteria, and to test the isolates’ ability to produce biofilm *in vitro*, and iv) describing the genotypic diversity of attached *E. coli*, to verify whether seaweeds host genetically-distinct types. To our knowledge, this is the first study performed so far to describe the association of *E. coli* with three common macroalgal genera (*Ulva*, *Sargassum* and *Undaria*) by adopting a temporal and spatial sampling strategy.

## Results

### *E. coli* abundance and distribution

*E. coli* was typically detected in all the investigated macroalgae, and the abundance varied according to the macroalga, sampling site and time (Fig. 2). During the low tide regime, abundance ranged from 5 ± 4 to 605 ± 83 CFU g^−1^ in *Ulva*, from 25 ± 21 to 3250 ± 636 CFU g^−1^ in *Sargassum* and from 6 ± 3 to 100 ± 71 CFU g^−1^ in *Undaria*, while ranged from 2 ± 1 to 416 ± 80 CFU mL^−1^ in the overlying water. The three-way ANOVA showed a significant interaction between macroalgae, sampling time and site on the *E. coli* abundance (Table S1). The SM site showed the highest abundance, with higher values observed on February 19^th^ and March 7^th^. The A site showed lower *E. coli* contamination in either macroalgae and water. The LL site, the farthest from the city center, showed the lowest concentration of attached *E. coli*, and a very low abundance in the water (as low as 1 CFU mL^−1^).

In the SM site, *Sargassum* generally hosted the highest abundances of *E. coli*, with values up to 6 times higher than the other macroalgae. Conversely, *Undaria* showed the lowest abundance. Similarly, in the A site, *Sargassum* showed the highest abundance of *E. coli*, while ranged from undetectable to 100 ± 71 CFU g^−1^ in *Undaria*. In both SM and A sampling sites, significant differences in *E. coli* abundances were observed according to sampling times in both water and macroalgae (ANOVA, p < 0.01). In the LL site, representing the less microbiologically polluted site, the higher *E. coli* concentrations were found in *Sargassum*.

The abundance of macroalgae-associated *E. coli* varied according to the tidal regime of the lagoon (Fig. S1), with higher values typically observed during low tide. The same temporal pattern was observed for *E. coli* abundance in water. Similar patterns were observed in the A site (data not shown). A significant, positive relationship between the *E. coli* abundance in macroalgae and water was observed (n = 101, R = 0.4169, p < 0.01).

### Phylotyping of *E. coli* isolates

Among the 378 isolates, 287 (76%) were identified as *E. coli* by the *uid*A gene amplification. The further characterization of confirmed isolates for their phylogroup of origin showed that the relative abundance of the phylogenetic groups changed among macroalgae and the overlying water (Fig. 3).

In the macroalgal samples, *Ulva* showed the highest percentage of isolates belonging to the B2 phylogroup (27%). *Sargassum* showed the highest fraction of isolates (18.5%) which could not be assigned to any phylogroup (defined “unknown”, according to the Clermont *et al*.^11^ method and requiring further MLST characterization), while *Undaria* showed the dominance of the A (46%) and C (24%) and the absence of B2 and E phylogroups. *Sargassum* hosted prevalently B1 isolates (27.8%), and comparable values of A and “unknown” (18.5% in both cases) isolates. The F phylogroup was observed in all the seaweed types. A low fraction (3.7 and 3.4%, respectively) of isolates from *Sargassum* and *Ulva* belonged to the cryptic clades I or II, and one isolate from *Sargassum* was assigned to the cryptic clade V.

As opposed to macroalgae, the phylogroups A and B1 were more frequently isolated in water (38 and 25%, respectively), followed by B2 (10.4%), F (6.7%) and all the other known groups (11.9%), including a small percentage of clade I or II (2.2%).

When all the isolates were grouped together (Fig. S2), independently from the isolation source (macroalga or water, hereafter defined as “habitat”), the largest fraction belonged to A (31%) and B1 (24.2%), followed by B2 (12.5%), F (7.8%), C (6.8%), D (3.2%) and E (2.9%) phylogroups. A percentage of isolates was “unknown” (8.9%), while a low fraction (ca. 3%) belonged to cryptic clades (I or II, and V).

### *E. coli* ability to grow on macroalgal extracts and produce biofilm

A subset of *E. coli* isolates, selected on the basis of their habitat of isolation or the sampling site, was used to perform two types of laboratory experiments (growth and biofilm production).

To test the potential of the isolates to multiply on macroalgae, we set-up a medium containing macroalgal extracts as the only source of carbon and nutrients. We tested 6 strains, two of which previously isolated from *Sargassum* (strain M340 and M349), two from *Ulva* (M223 and M360), one from *Undaria* (strain M306) and one from seawater (M378). Each strain was tested on all three macroalgal-based media and at three different temperatures, i.e., 10 °C, 20 °C and 37 °C. All the macroalgal extracts supported the growth of the tested strains under the different temperatures (Fig. 4A–C, Fig. S3A-F), suggesting that *E. coli* can grow on macroalgae as the only source of carbon and nutrients. However, the growth was not dependent upon the habitat of isolation. At 10 °C and 20 °C, the tested strains showed an overall higher and faster growth on *Sargassum* extract and, only in some instances, isolates grew better on the extract prepared with the macroalga of isolation (Fig. S3B-C). At 37 °C, *E. coli* strains grew faster when compared with 10 °C and 20 °C, by reaching the stationary phase within 4–6 hours, and generally showed higher OD_600_ values in the *Ulva* extracts. However, these patterns were not consistently observed, and other isolates showed comparable growth on media prepared with two or even three different macroalgae (Fig. 4A–C, Fig. S3A,C). This was especially true at 20 °C while at 10 °C, for most of the tested strains, a higher variability in OD_600_ values was observed among algal extracts (Fig. S3A-C). The strain isolated from seawater demonstrated ability to grow on all macroalgal media at all tested temperatures (data not shown).

A second subset of isolates, including those previously used for growth experiments, was tested for the ability to form biofilm at 20 °C and 37 °C. We tested 21 isolates from all three seaweed types (1 from *Undaria*, 7 from *Sargassum*, 8 from *Ulva*) and the overlying seawater (5 isolates). The isolates were variably able to form biofilm, ranging from no production to strong production (Fig. 5). At 20 °C, several *E. coli* isolates showed capability to produce biofilm, evident from the large percentage of strong (12.5%), moderate (6.25%) and weak (68.75%) producers, while only 12.5% of isolates did not form biofilm. At 37 °C, a high but lower percentage of macroalgal isolates was able to produce biofilm (6% strong, 13% moderate and 31% weak producers), while the remaining 50% of isolates did not form biofilm. Most isolates from water did not produce biofilm (60%), and the remaining isolates were weak or moderate producers (20% in both cases). Conversely, no strong biofilm producers *E. coli* were isolated from water. Similarly, for the water-isolated strains, all non producers isolates observed at 37 °C became weak biofilm producers (for a total of 80%) at 20 °C.

### Genetic diversity of *E. coli* isolates

Ninety-nine isolates, selected on the basis of their habitat of isolation and the sampling event, were typed by an improved high-resolution RAPD (Random[ly] Amplified Polymorphic DNA) protocol. This protocol allowed discriminating differences as small as 3–5 base pairs (for fragments ≤ 500 bp), a value much higher than obtained on conventional RAPD protocols on agarose gel. Replicated RAPD analyses performed on the same isolates yielded similarity percentages from 98 to 100% (data not shown). We subsequently used the most conservative value (98%) as value above which two isolates are considered as clones.

The dendrograms divided per habitat and the overall dendrogram are reported in the Supplemental Material (Fig. S4-S5 respectively). The results highlighted a wide genetic diversity of *E. coli* attached to macroalgae, with very few clonal isolates (only observed in *Ulva*, e.g. M17–M56, and M212–M223) and a large number of unique genotypes. Similarity values ranged from ca. 6 to 88% in *Sargassum*, from ca. 14 to 100% in *Ulva* and 8 to 88% in *Undaria*. The diversity of *E. coli* isolated from water samples was similarly very high but, as opposed to seaweeds, showed higher frequency of clonal clusters (e.g. M210–M282–M332).

When all isolates were grouped together (Fig. S5), the ANOSIM analysis showed significant differences in the genetic diversity according to the habitat (macroalgae vs. water; Global R = 0.148, significance level < 0.001), with significant differences observed between *Undaria* and water (Global R = 0.308, significance level < 0.001), *Sargassum* and water (Global R = 0.192, significance level < 0.001) and *Ulva* and water (Global R = 0.102, significance level < 0.001) and no differences among macroalgae (for all comparisons, ns). Conversely, the ANOSIM did not reveal differences in the distribution of *E. coli* genotypes according to the sampling time or site (ANOSIM, ns). Typically, isolates from the same macroalgae did not group with each other, showing a high genetic diversity even within the same sample. However, identical clones were sometimes found associated with different macroalgae from the same site and sampling event (M237–M223, and M162–M193). Similarly, the same clones were occasionally shared by macroalgae and the water collected in a different site (e.g. M234–M32 and M304–M282–M332). Some genotypically identical isolates were observed in the water collected in different sites (M210–M282–M332).

## Discussion

A large body of evidence demonstrated that *E. coli* can persist outside the hosts, likely due to its high versatility and genetic diversity^42^. Studying the fate of *E. coli* in non-enteric habitats, and identifying environmental reservoirs, is crucial to evaluate the potential acquisition of virulence or antibiotic-resistance properties, leading to infections by pathogenic *E. coli* (such as the O157:H7 serotype) suspected to evolve in the environment^43^,^44^. Recent studies identified reservoirs such as beach sands^2^,^45^, marine sediments and wetlands^3^,^4^,^46^. Freshwater macroalgae, such as *Cladophora*, can harbor FIB in lakes^24^,^27^,^29^,^47^. However, similar studies have not been performed in coastal marine and transitional environments, nor have investigated the role of common macroalgae, such as those belonging to the genera *Ulva*, *Sargassum* and *Undaria*, as *E. coli* sources.

We demonstrated that *E. coli* can be associated with live macroalgae other than *Cladophora*. The abundance was highly variable on both spatial and temporal scales. The brown algae *Sargassum muticum* and *Undaria pinnatifida* are invasive species, typically found in temperate to cold-temperate habitats, while green *Ulva* species have a nearly ubiquitous distribution^31^. These algal genera display different features potentially influencing bacterial adhesion and survival, including life cycle, light requirement, biochemical composition^48^ and the surface-to-volume (S/V) ratio. Due to its branching morphology, *Sargassum* offer higher S/V ratio than the other seaweeds, characterized by a laminar morphology, which may explain the higher concentrations observed in *Sargassum*. Because of the wide metabolic and genetic versatility and ability to colonize diverse macroalgae, it is likely that also other macroalgal genera may serve as unidentified sources or reservoirs for *E. coli*. The only studies available for comparison refer to the macroalga *Cladophora*, and have been performed across the Lake Michigan (USA). The authors report a similar range of abundance, and comparable spatial and temporal variability in *E. coli* abundance in live and floating macroalgae^21^,^27^. Olapade *et al*.^49^ reported up to 60,000 CFU 100 g^−1^ in *Cladophora* mats in lake shores, while Imamura *et al*.^30^ reported that dead macroalgae (algal wracks) stranded in California beaches hosted > 4 log CFU dry g^−1^.

The *E. coli* abundance in water showed values comparable to previous findings in the area^50^. The presence of large populations of *E. coli* is clearly linked to the lack, in the historical centre of Venice, of modern depuration plants and the input of almost untreated wastes^51^, despite the efforts in place to provide the city with more efficient sewage treatment plants. The abundance in water changed according to the tidal regime, with higher abundances during low tide. This pattern is possibly driven by the water exchanges between the lagoon and the sea during the high tide, favouring inputs of less contaminated water from the sea. The water circulation in the lagoon is mainly driven by tides (up to 1-m excursion) and wind, and the amount of salt water flowing in and out at each tidal cycle amounts to one third of the lagoon volume^52^. This pattern was reflected in the macroalgae, which showed higher bacterial abundance during low tide. Moreover, the abundance of algal-attached *E. coli* was positively related with the abundance in the overlying water. Similar studies reported that *E. coli* concentrations in *Cladophora* and lake water were correlated^27^,^53^. Englebert *et al*.^54^ reported that *E. coli* abundance was higher in the water above *Cladophora* mats than in water far away, suggesting release from mats due to wind and wave action. Taken together, these results suggest that, while *E. coli* may not be a typical member of the epiphytic bacterial flora in non-polluted environments^22^, it can colonize the macroalgal surface in chronically-polluted areas.

Phylogenetic analysis highlighted the presence of all known phylogroups of *E. coli sensu stricto*, including the commensal A and B1, the potentially pathogenic B2 and D (causing extra-intestinal infections)^55^ and the recently described C (distinct but closely related to B1)^56^,^57^, E (formerly a set of unassigned strains, of which the O157:H7 is the best known member)^11^ and F^57^,^58^. These results are the first on the occurrence, using the recent method by Clermont *et al*.^11^, of C, E and F phylogroups in association with macroalgae and, more broadly, in the aquatic environment. Studies carried to investigate the prevalence of phylogroups have mostly addressed estuarine and freshwater environments^59^,^60^ rather than marine ones^3^. We also highlighted potential phylogroup-macroalgal associations, as in the case of *Sargassum* being the only seaweed hosting all phylogroups and the cryptic clade V. Conversely, neither cryptic clades nor B2, E and “unknown” isolates were observed in *Undaria*. This non-random distribution of phylogroups among seaweeds let hypothesize that the different characteristics of the algae may select for certain phylogroups. However, this hypothesis needs to be tested on a larger number of isolates. Phylogrouping analyses highlighted presence of a low percentage of cryptic clades. Limited information is available about cryptic lineages in aquatic environments. They are likely representing environmentally adapted *Escherichia* lineages^12^,^13^ and seem to be more abundant in certain environments, such as sediments^9^,^12^. We suggest that the macroalgal habitat may favor the evolution of some *Escherichia* cryptic clades, which deserves further investigations.

Laboratory experiments performed at *in situ* and 37 °C temperatures showed that the three macroalgae can, under laboratory conditions, support the growth of *E. coli* as only source of carbon and nutrients, and that several strains can form biofilms. A similar *in vitro* ability was demonstrated for fecal *Cladophora* isolates^24^. These findings emphasize the role of seaweeds as potential *E. coli* sources in coastal areas. The ability of different isolates to grow on different algae, and similarly the ability of *E. coli* isolated from water to grow on macroalgal extracts, suggests that *E. coli* can adapt to grow on a variety of organic substrates, and pass from water to the macroalgal habitat and viceversa. Finally, the ability of several macroalgal isolates to produce biofilm at different temperatures suggests that a biofilm lifestyle may be common among attached *E. coli* and raises questions about the potential virulence of environmental *E. coli*. In the environment, the biofilm lifestyle may confer several ecological advantages, such as higher resistance to predators or UV radiations, and increased resource availability^20^. Similarly to our findings, Moreira *et al*.^61^ reported superior ability to form biofilm by *E. coli* isolated from freshwater periphyton in comparison with human strains, suggesting that the biofilm lifestyle increased persistence in the environment.

RAPD analyses highlighted a high genotypic diversity of macroalgae-associated *E. coli*, consistently to previous results^47^. Our similarity values were in a range similar to those reported, using the REP-PCR fingerprinting technique, in *Cladophora* mats^28^,^47^. Despite the ANOSIM test revealed significant differences in the distribution of *E. coli* genotypes among macroalgae and water, we didn’t find clear clustering of isolates according with time, site or macroalga. This high genetic heterogeneity suggests that macroalgae are not selecting for specific genotypes, and that the majority of attached genotypes are likely transient members, whose large genotypic diversity reflects the wide variety of sources of fecal contamination from the city, which is visited yearly by more than 10 millions of tourists from worldwide. This agrees with Byappanahalli *et al*.^24^, who suggested that the association between *E. coli* and macroalgae may be not algal-specific, and is supported by our experimental findings that different isolates grow on all the macroalgal types. However, at the same time, we found that some genotypically identical isolates were associated to diverse macroalgae collected from different sites. This would suggest that at least some genotypes can be consistently associated with macroalgae, as later reported by Byappanahalli *et al*.^47^ in *Cladophora* on a larger dataset, and that the association may be advantageous for survival of those genotypes. Further genotypic analyses on a larger dataset will allow to clarify this issue.

This study is of relevance for the city of Venice, where high macroalgal biomasses are present in certain periods of the year. The waters around the city receive large loads of FIB, and are not suitable for bathing. The presence of FIB determines human health hazards through unusual routes of exposure, such as aerosolization of polluted waters by the frequent boats traffic (which may lead to inhalation of pathogens) or the recurrent floods (“acqua alta”) inundating streets and houses and determining waterborne disease exposure scenarios^36^. This large macroalgal source of FIB represents an additional, so far unrecognized, health hazard and poses extra questions about the spread of waterborne infectious diseases in the area. Macroalgae could serve as a continuous source of fecal and potentially pathogenic bacteria, likely able to alter the water quality of surrounding coastal areas.

We conclude that cosmopolitan and invasive macroalgae can serve as environmental sources or reservoirs of *E. coli*, including potentially pathogenic strains. Given the wide geographic diffusion of the studied macroalgae and the *E. coli* ability to colonize different seaweeds, this phenomenon is likely to occur in other coastal cities worldwide and can potentially alter the water quality of shorelines, deserving investigations from either the human health and ecological perspectives.

## Methods

### Study area and sampling activities

**Figure 1:**
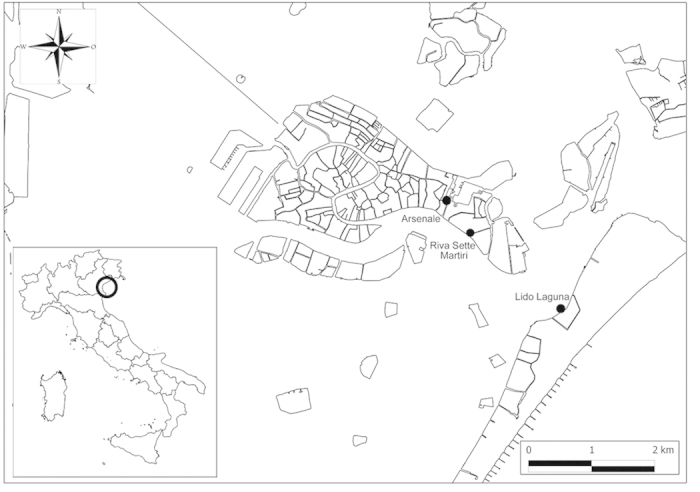
Study area and sampling sites. The study area and the sampling sites located around the city of Venice (Italy). The sites were selected on the basis of the presumptive level of fecal contamination. The sites SM and A were located closer to the historical centre of Venice, thus under closer proximity to the sources of fecal bacteria. The LL site was located in the Lido island, at higher distance from the city centre and closer to the Lido inlet, which exchanges water with the sea, thus representing a potentially less contaminated site. The latitude and longitude of the three sites were: 45°25’48.77”N, 12°21’16.56”E (site SM), 45°26’2.24”N, 12°21’0.32”E (site A), 45°25’10.21”N, 12°22’26.38” E (site LL). The map was created with QGIS v2.2.0 software.

**Figure 2:**
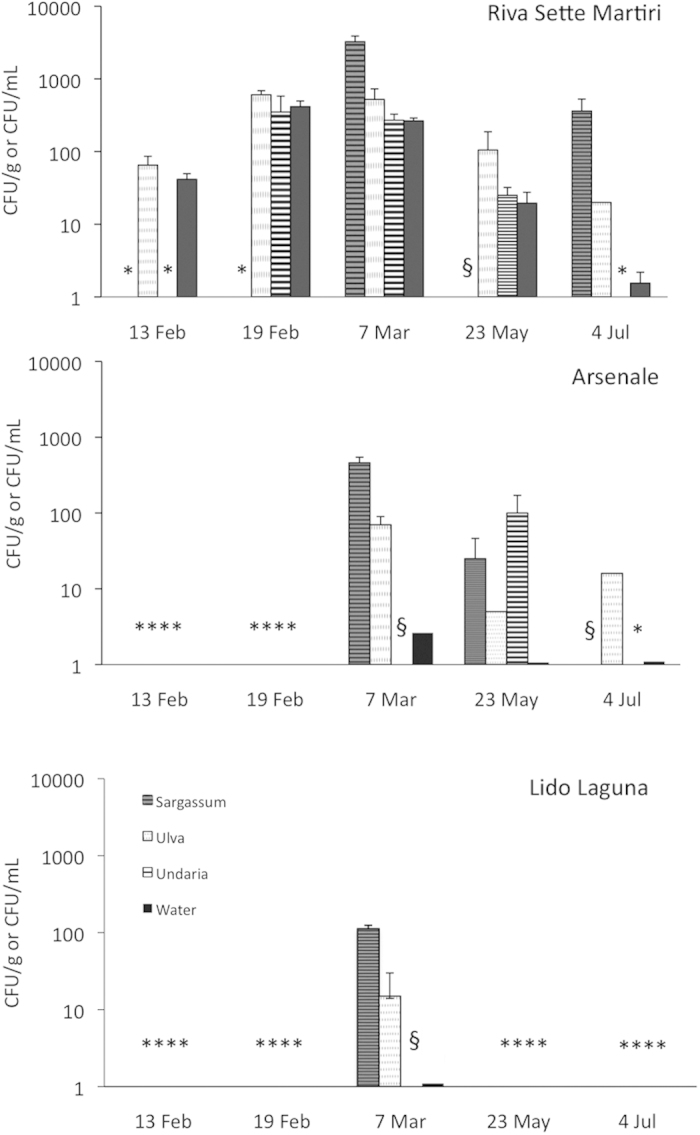
Abundance of ***E. coli*** in the three sampling sites during the low tide regime. Data represent average ± standard error and are expressed as CFU g^−1^ (for macroalgae) or mL^−1^ (for water). * = data not available due to the absence of the macroalga in the sampling site; § = no *E. coli* growth was observed.

**Figure 3:**
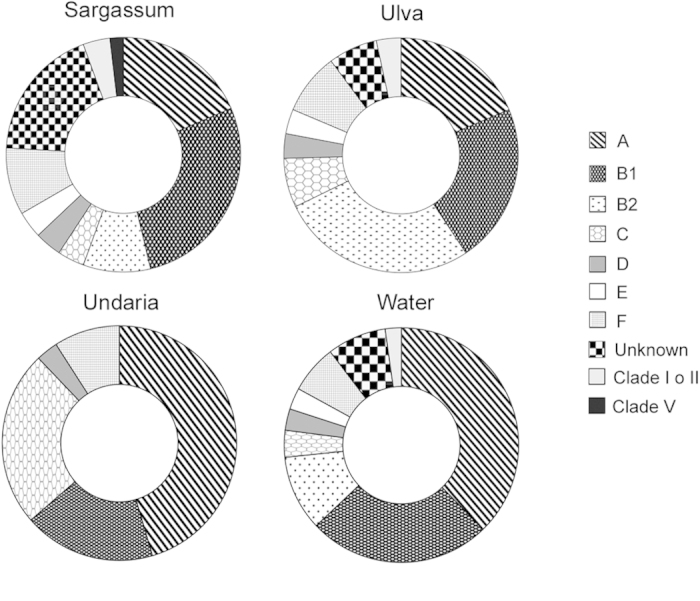
Distribution of the ***E. coli*** phylogroups in the macroalgae and water samples. Data are expressed as percentage (%).

**Figure 4:**
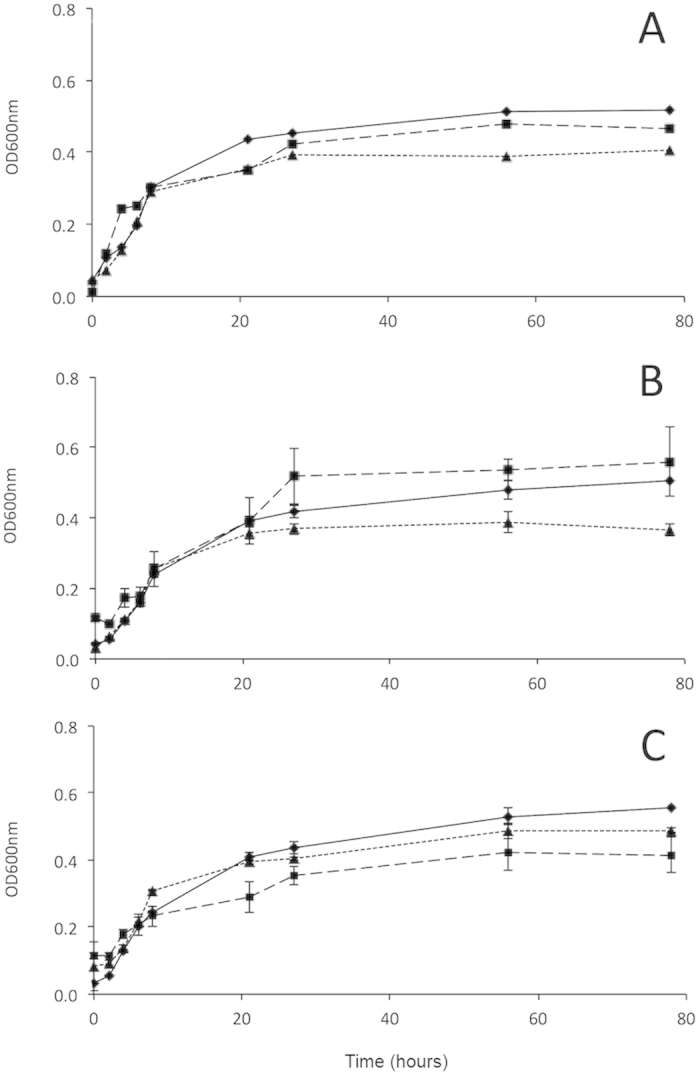
Growth curves on macroalgal extracts at 20 °C for representative *E. coli* isolates. A = strain #223 isolated from *Ulva*; B = strain #349 isolated from *Sargassum*; C = strain #306 isolated from *Undaria*. Triangles refer to the growth on the *Undaria* medium, while rhombuses to the growth on *Sargassum* and squares on *Ulva*. The data are averages ± standard errors of the averages. OD600nm = optical density at 600 nm.

**Figure 5:**
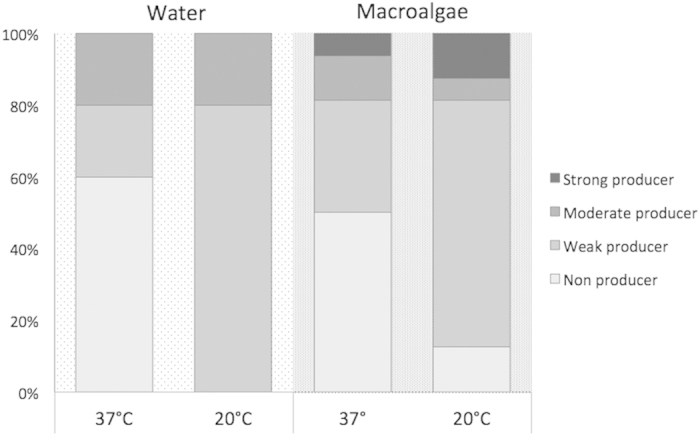
Biofilm producer *E. coli* strains. Percentages of biofilm producers *in vitro* among the *E. coli* isolated from macroalgae and the overlying water at 37 °C and 20 °C.

*Sargassum muticum* (Yendo) Fensholt, *Undaria pinnatifida* (Harvey) Suringar and *Ulva* species and overlying water were collected aseptically from three sites (Sette Martiri – SM, Arsenale – A, Lido Laguna – LL; Fig. 1 and S6). Samples were collected typically once per month, with the exception of February when samples were collected twice to firstly evaluate the differences under a shorter temporal scale (February 13^th^ and 19^th^, March 7^th^, May 23^th^ and July 4^th^ 2013). Samples were collected twice during both low and high tide, except on February. Live macroalgal samples (attached to substrates such as canal embankments or docks) were collected and analysed (two replicates) using sterilized tweezers to avoid contamination. Seaweeds were put into sterile containers, maintained at 4 °C and analysed within 2–6 hours from sampling. One liter of water overlying the macroalgae was put into sterile bottles and stored at 4 °C in the dark until processing as above.

### Abundance of *E. coli* in macroalgae and water

Aliquots of 1 gram of macroalgae were transferred into sterile tubes and suspended into 9 mL of sterile 0.8% NaCl. For bacterial detachment, similarly to previous studies on macroalgae^24^,^27^,^47^, samples were vigorously shaken and sonicated (3 times, 1 minute each cycle with 30 sec intervals within cycles). Serial ten-fold dilutions (1:10 and sometimes 1:100) were performed. Aliquots of 1 mL of the supernatant and the obtained dilutions were filtered and analysed using the Membrane Filtration technique as described previously^3^. Filters were plated onto mFC agar plates (Biolife) and incubated for 24 hours at 44.5 °C. For water samples, 1, 10 and 100 mL aliquots were filtered (three replicates) and the filters plated and incubated as above. Blue colonies were considered as presumptive *E. coli* and isolated for further analyses. The abundance of presumptive *E. coli* was reported as CFU per gram of wet macroalgae or mL of water.

### Identification of *E. coli* isolates

Well-separated presumptive *E. coli* colonies were streaked on agar for isolation and cultured overnight in Tryptone Soy Broth (Biolife) with 0.3% Yeast Extract (Biolife). Lysates of overnight cultures were used as a DNA template in all PCRs protocols in this study. Identification of presumptive *E. coli* colonies (n = 378) was performed by amplifying the *uid*A gene^62^. PCR products were separated by agarose gel electrophoresis (1%) and visualized with GelRed (Biotium).

### Determination of phylogenetic groups and cryptic clades

The 287 *Escherichia* isolates were analyzed using the method by Clermont *et al*.^11^, which allows to assign isolates to the seven phylogroups (A, B1, B2, C, D, E and F) or to the cryptic *Escherichia* clades. Isolates belonging to cryptic clades I or II and III, IV or V were confirmed by the Clermont *et al*.^57^ protocol. PCR products were separated by agarose gel electrophoresis (2%) and visualized with GelRed (Biotium).

### Growth of *E. coli* on macroalgal extracts

Macroalgal extracts were used as growth medium to investigate the ability of *E. coli* to multiply using algae as only carbon and nutrient source. *Ulva* spp., *Sargassum muticum* and *Undaria pinnatifida* samples were collected and put at −80 °C for 24 hours to kill the epiphytic microbes. This procedure was preferred to other procedures (e.g. autoclaving), which would have altered the algal chemical composition and destroyed important macromolecules for bacterial growth. *E. coli* strains were inoculated in algal extracts at 10 °C, 20 °C and 37 °C. The first two temperatures were chosen as representative for the late winter (February to April) and spring-summer (May to July) periods on the basis of periodical measurements performed by the local municipality (data not shown). The growth curve at 37 °C was performed as representative of the optimal growth temperature for *E. coli*. The efficiency of the freezing procedure was tested on uninoculated macroalgal extracts, incubated at the three different temperatures. The tests revealed that extracts did not show growth over a 80 hours period (for the tests carried out at 10 °C and 20 °C), while contamination was detected after about 8 hours when testing the growth at 37 °C. This suggested to perform shorter incubations (e.g. until 6 hours) for experiments at 37 °C to ensure measuring the growth of only the studied strains. To prepare the algal-made medium, each algae was put into a sterile tube and added with sterile 0.8% NaCl (1:10 w/v). This mixture was whisked aseptically with an electric blender for 2 minutes. The supernatant was filtered through a sterile Whatman #3 membrane (to eliminate large particles) and the crude algal extract collected into a sterile bottle. The growth curves for the isolates (six *E. coli* tested in triplicate) were obtained by measuring spectrophotometrically the optical density (λ = 600 nm) of extracts inoculated with 1% (v/v) of a single strain overnight broth culture (OD_600_ = 0.9 - 1) and incubated aerobically at 37 °C up to 6 hours.

### Biofilm production *in vitro*

The biofilm production test was performed as described by Vignaroli *et al*.^4^ and references therein. Bacterial cells were grown overnight in Luria-Bertani broth (LB) (Oxoid) supplemented with 1% glucose (LBG) at 37 °C and diluted to an OD_625_ of 0.1. These suspensions (0.2 mL) were incubated overnight at 20 °C and 37 °C in 96-well polystyrene microtiter plates (Falcon, Becton Dickinson Labware). The broth cultures were aspirated and the wells washed three times with 0.2 mL of phosphate-buffered saline (PBS). Microtiters were dried at 60 °C for 1 hour, and stained with 0.1 mL of Hucker’s Crystal Violet (CV) solution (10% crystal violet in 20% ethanol containing 1% ammonium oxalate) for 10 min. CV was aspirated, and wells were washed with sterile water. CV was extracted from adhering bacterial cells by ethyl alcohol/acetone (80:20 v/v), and the OD_690_ was measured using a microplate reader (Thermo Electron Corporation, Madison, WI). Each assay was performed in triplicate. Strains were classified as non producer (OD ≤ ODc), weak producer (ODc < OD ≤ 2 × ODc), moderate producer (2 × ODc < OD < 4 × ODc) or strong producer (OD > 4 × ODc). The OD cutoff (ODc) was defined as 3 standard deviations above the mean OD of the negative control represented by uninoculated wells containing LBG. The strong biofilm producer *S. epidermidis* ATCC 35984 was used as the positive control.

### High-resolution genotyping

Ninety-nine *E. coli* strains were genotyped by using RAPD (Random[ly] Amplified Polymorphic DNA) PCR, and the amplicons analyzed using the high-resolution Qiaxcel Advanced capillary electrophoresis System (Qiagen). We selected the RAPD technique which, despite certain limitations, is a useful tool to provide initial insights on the genotypic diversity of large numbers of isolates^63^,^64^,^65^. PCR were performed as described by Regua-Mangia *et al*.^66^ and Luna *et al*.^3^, using 1 μL of bacterial lysate. Amplification products were purified and analysed using Qiaxcel. A dendrogram was obtained by analyzing electrophoretic peaks on Bionumerics v7.0 software (Applied Maths), using the Dice similarity coefficient and UPGMA (Unweighted Pair Group Method of Averages) as clustering method.

### Statistical analyses

Differences in *E. coli* abundance between macroalgae were assessed using a three-way analysis of variance (ANOVA) carried out on the low-tide abundance dataset. The analysis treated the factor site (S, 2 levels: A and SM sites) as fixed, time (T, 5 levels: February 13^th^, February 19^th^, March 7^th^, May 23^th^ and July 4^th^) as fixed and crossed with S, and habitat type (H, 4 levels: *Ulva*, *Undaria*, *Sargassum* and water) as random and orthogonal. The LL site was excluded due to the low number of samples. When significant differences (p < 0.05) were observed for fixed factors, a post-hoc Student-Newman-Kuels’ test (SNK) was performed. Prior to the analysis, the homogeneity of variance was checked using the Cochran’s test on appropriately transformed data, whenever necessary. If the transformation did not allow to obtain an homogeneous variance, a more conservative level of significance was considered. ANOVA and SNK tests were carried out using GMAV (University of Sydney). Differences in the genotypic diversity of *E. coli* isolates from different habitats were assessed on the RAPD dataset using the analysis of similarity (ANOSIM) tool based on a Bray-Curtis similarity matrix. The presence of statistical differences between samples is indicated by a significance level at least p < 0.05. The ANOSIM analysis was performed using the PRIMER 6 + software (http://www.primer-e.com/).

## Additional Information

**How to cite this article**: Quero, G. M. *et al*. Understanding the association of *Escherichia coli* with diverse macroalgae in the lagoon of Venice. *Sci. Rep*. **5**, 10969; doi: 10.1038/srep10969 (2015).

## Supplementary Material

Supplementary Information
