# Supplementary material for: Understanding the association of Escherichia coli with diverse macroalgae in the lagoon of Venice
Source: Sci Rep. 2015 Jun 4;5:10969. doi: 10.1038/srep10969 (PMC4455311; doi:10.1038/srep10969)

Supplementary Information

Understanding the association of *Escherichia coli* with diverse macroalgae in the lagoon of Venice

Grazia M. Quero[1], Luca Fasolato[2], Carla Vignaroli[3] ,Gian Marco Luna[1,*]

[1]Institute of Marine Sciences (CNR – ISMAR), National Research Council, Venezia, Italy,
[2]Department of Comparative Biomedicine and Food Science, University of Padova, Italy,
[3]Department of Life and Environmental Sciences, Polytechnic University of Marche, Ancona, Italy

[*]Corresponding author
Gian Marco Luna
National Research Council – Institute of Marine Sciences (CNR – ISMAR)
Castello 2737/f – Arsenale Tesa 104
30122 Venezia, Italy
Tel. +39 041 2407993
email: gianmarco.luna@ve.ismar.cnr.it

Table S1.
Figures S1 – S2 – S3 – S4 – S5 – S6.

Supplementary Table S1. Results of the three-way ANOVA test on the abundance of *E. coli* in the study area. SS= sum of squares; DF = degrees of freedom; MS = mean square; F = statistic F. Reported are also the results of the Cochran's test performed prior to the ANOVA analysis to test the homogeneity of variance.

Transform: Ln(X+1)

Cochran's Test

C = 0.3267 (P < 0.01)

**Source**	**SS**	**DF**	**MS**	**F**	**P**
SITE	173.6255	1	173.6255	17.98	0.0240
TIME	183.7927	4	45.9482	3.45	0.0428
HABITAT	28.6240	3	9.5413	34.52	0.0001
SI × TI	64.2726	4	16.068	1.63	0.2311
SI × HA	28.9691	3	9.6564	34.93	0.0001
TI × HA	160.0020	12	13.3335	48.24	0.0001
SI × TI × HA	118.5239	12	9.8770	35.73	0.0001
RES	22.1131	80	0.2764		
TOT	779.9229	119			

Supplementary Figure S1. Abundance of *E. coli* (average ± standard error) in the Sette Martiri sampling site during low and high tide regime. Data are expressed as CFU g^{-1} (for macroalgae) or CFU ml^{-1} (for water). § = no *E. coli* growth was observed. The *E. coli* abundance in water on the 23 May during high tide is 0.06 ± 0.05 CFU/ml.

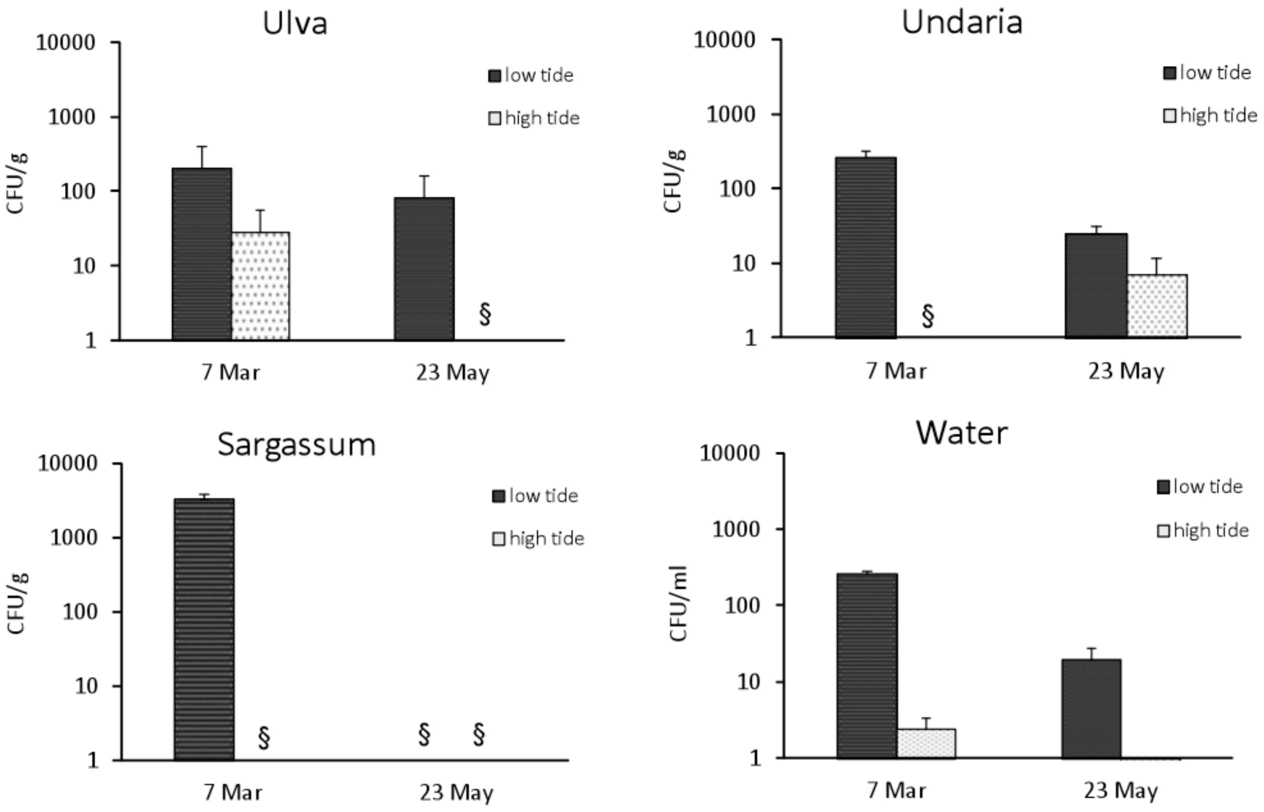

Supplementary Figure S2. Cumulative distribution (expressed as percentage, %) of the *E. coli* phylogroups in the entire set of isolates (macroalgae and water).

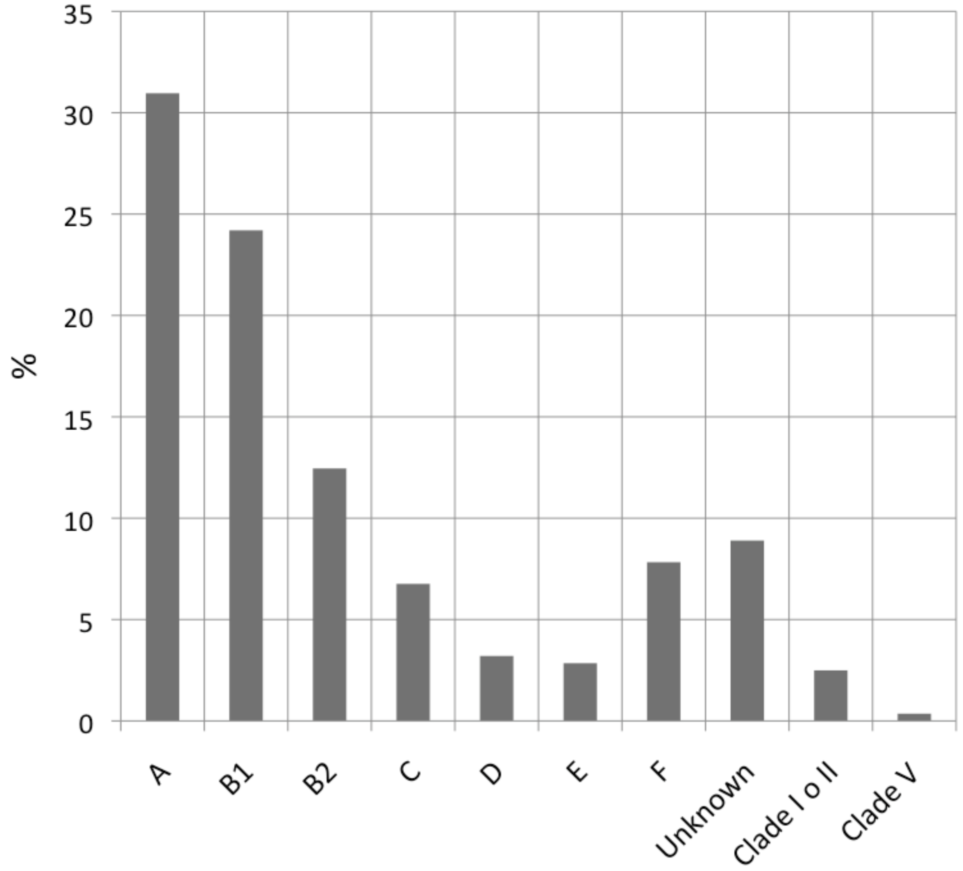

Supplementary Figure S3. Growth curves on macroalgal extracts for representative *E. coli* isolates. A and D= strain #223 isolated from *Ulva*; B and E = strain #349 isolated from *Sargassum*; C = strain #306 isolated from *Undaria*, F = strain #360 isolated from *Ulva*. Panel A, B and C refer to *E. coli* isolates growth at 10°C; panels D, E and F to *E. coli* growth at 37°C. Triangles refer to the growth on the *Undaria* medium, while rhombuses to the growth on *Sargassum* and squares on *Ulva*. The data are averages ± standard errors of the averages. OD600nm = optical density at 600 nm.

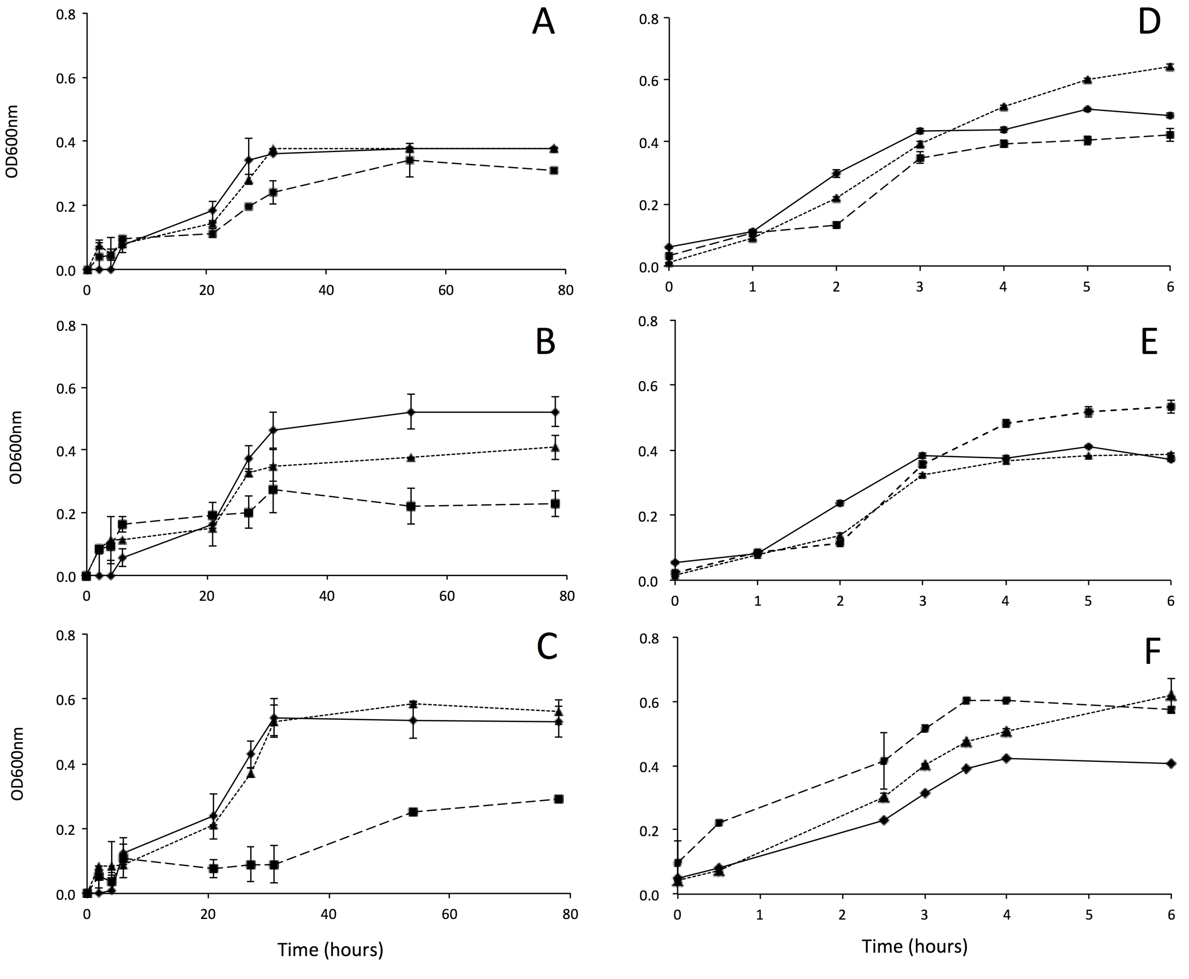

Supplementary Figure S4. Dendrograms showing the genetic diversity of the *E. coli* typed in this study, as divided per habitat (the three macroalgae and the overlying water). The top axis shows the percent similarity among the genotypes, as indicated by the Dice correlation coefficients. Numbers on the left side indicate the isolate's reference number.

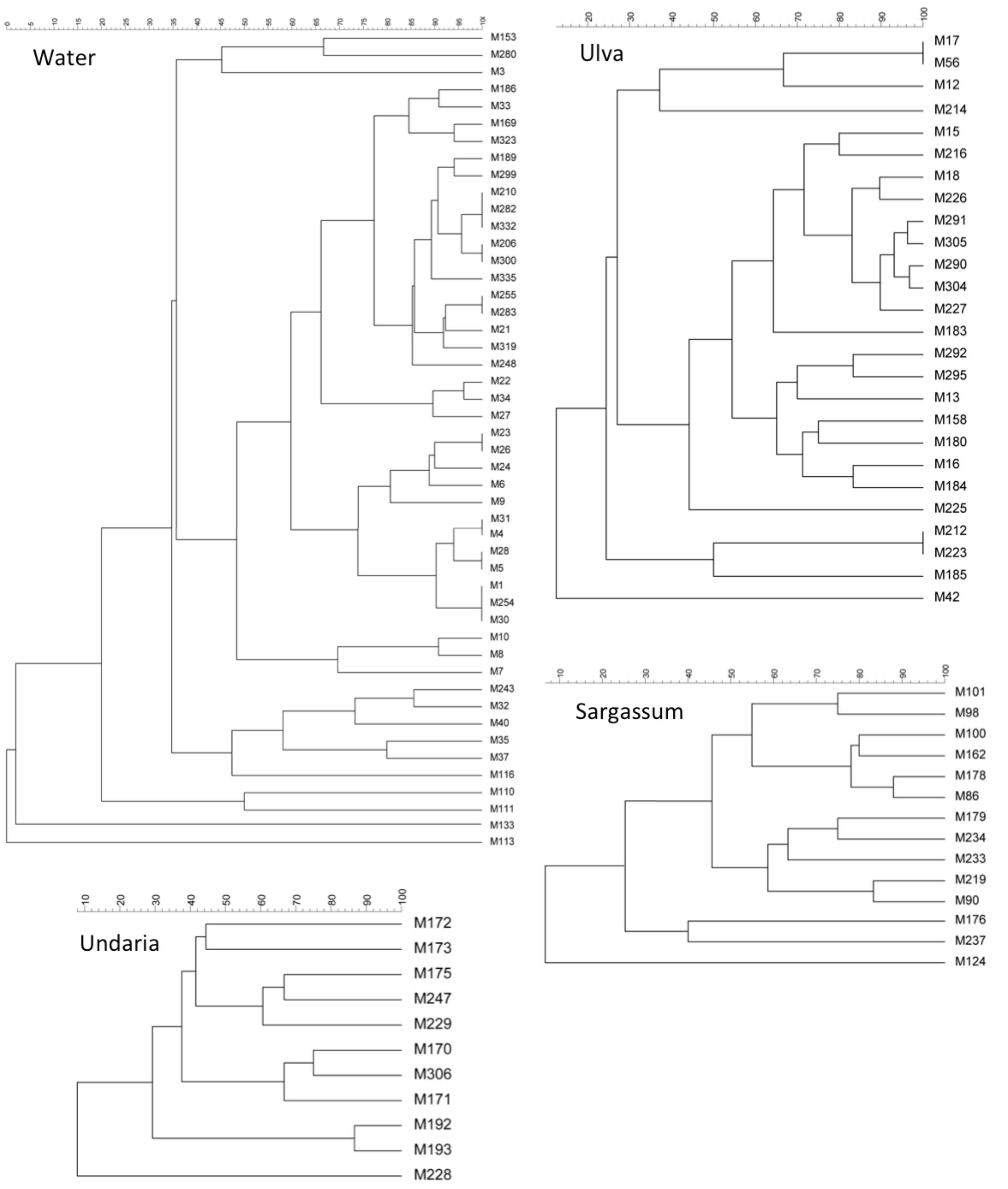

Supplementary Figure S5. Dendrogram showing the genetic diversity of all *E. coli* isolates typed in this study. The top axis shows the percent similarity among the genotypes, as indicated by the Dice correlation coefficient. Numbers on the right side indicate the isolate reference number. Reported on the right are also the habitat type (*Undaria*, *Ulva*, *Sargassum* or water), the sampling time (Feb = February; Mar = March; or May) and the sampling site.

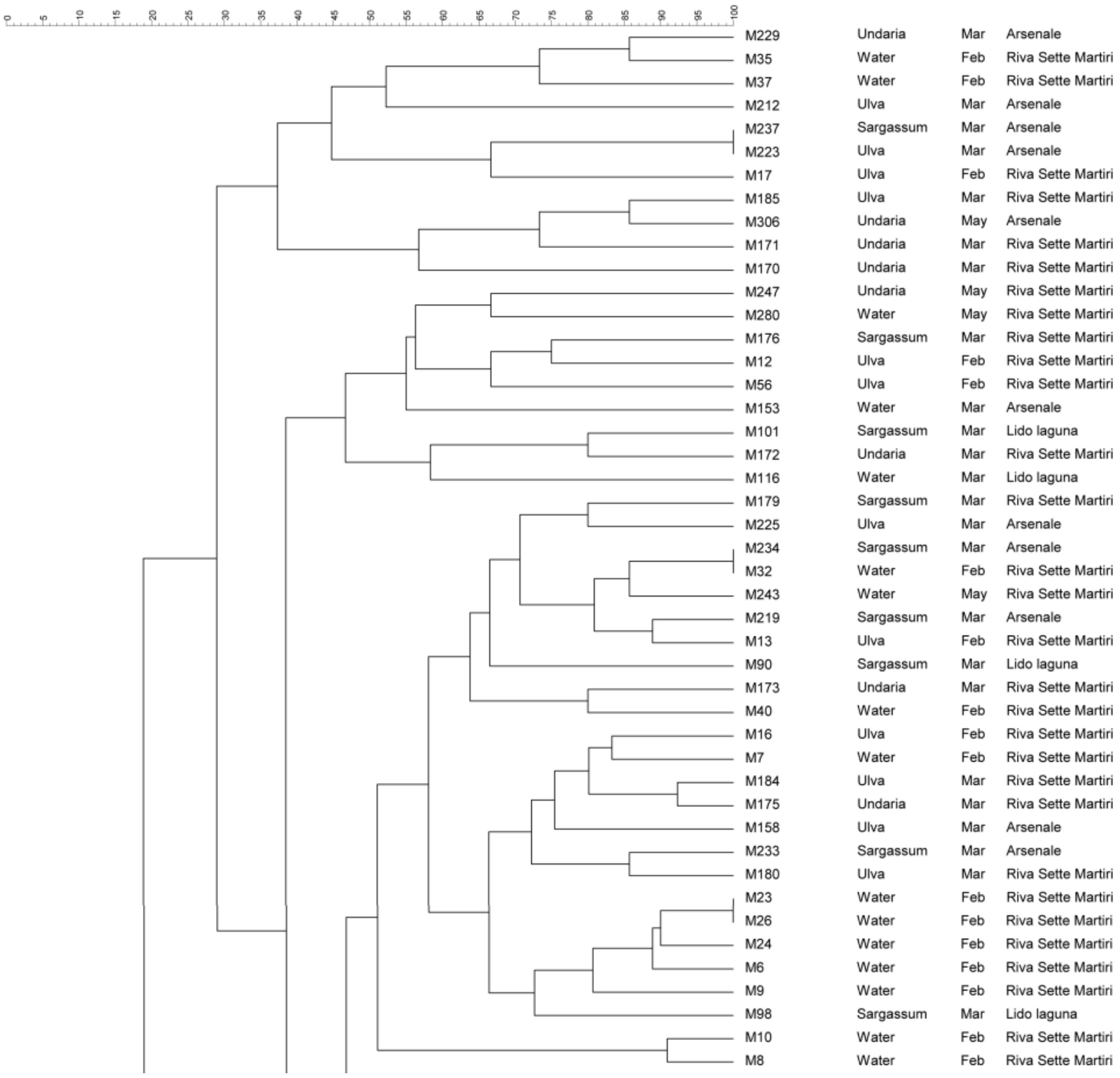

(continued on the next page)

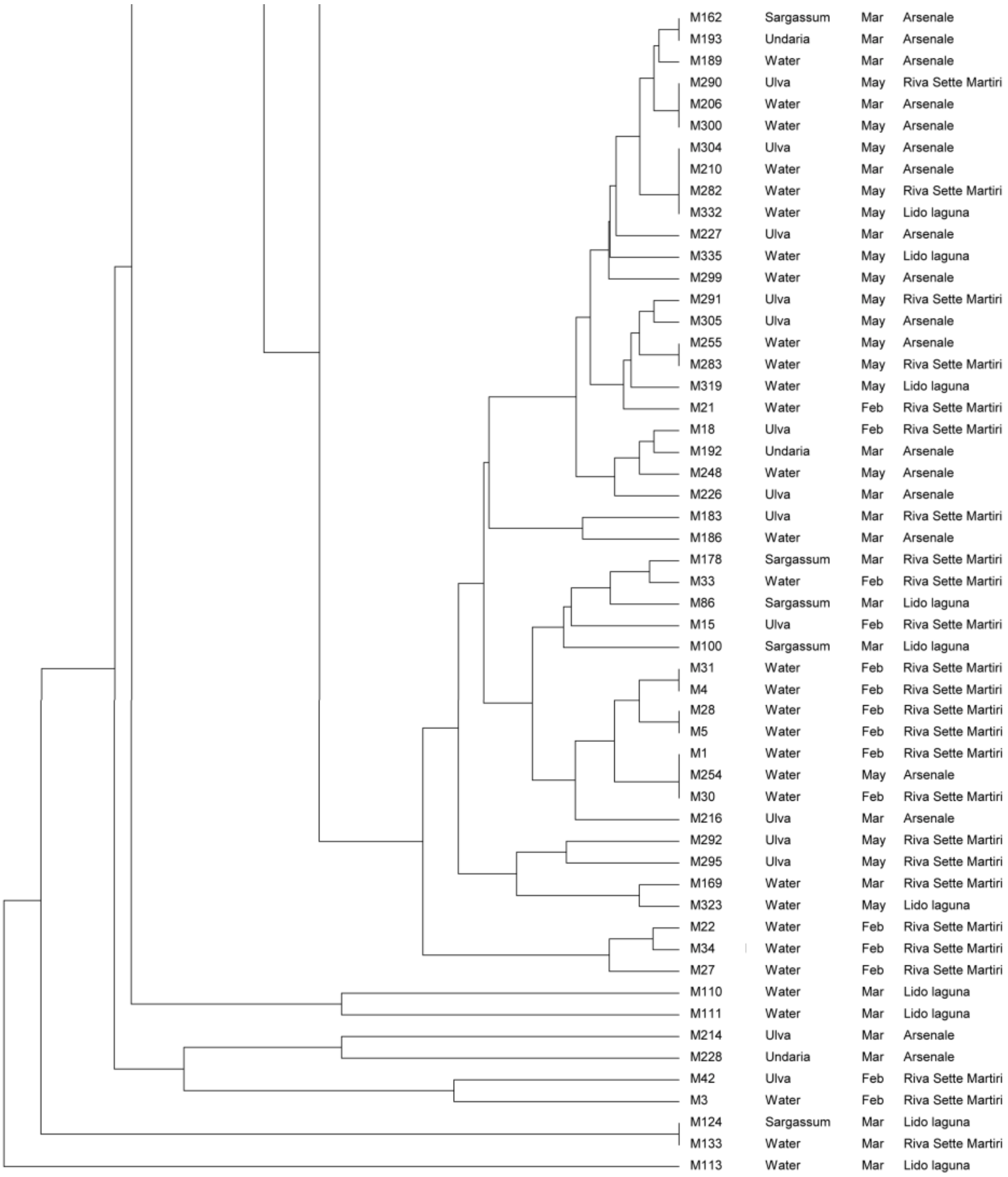
M162 Sargassum Mar Arsenale
M193 Undaria Mar Arsenale
M189 Water Mar Arsenale
M290 Ulva May Riva Sette Martiri
M206 Water Mar Arsenale
M300 Water May Arsenale
M304 Ulva May Arsenale
M210 Water Mar Arsenale
M282 Water May Riva Sette Martiri
M332 Water May Lido laguna
M227 Ulva Mar Arsenale
M335 Water May Lido laguna
M299 Water May Arsenale
M291 Ulva May Riva Sette Martiri
M305 Ulva May Arsenale
M255 Water May Arsenale
M283 Water May Riva Sette Martiri
M319 Water May Lido laguna
M21 Water Feb Riva Sette Martiri
M18 Ulva Feb Riva Sette Martiri
M192 Undaria Mar Arsenale
M248 Water May Arsenale
M226 Ulva Mar Arsenale
M183 Ulva Mar Riva Sette Martiri
M186 Water Mar Arsenale
M178 Sargassum Mar Riva Sette Martiri
M33 Water Feb Riva Sette Martiri
M86 Sargassum Mar Lido laguna
M15 Ulva Feb Riva Sette Martiri
M100 Sargassum Mar Lido laguna
M31 Water Feb Riva Sette Martiri
M4 Water Feb Riva Sette Martiri
M28 Water Feb Riva Sette Martiri
M5 Water Feb Riva Sette Martiri
M1 Water Feb Riva Sette Martiri
M254 Water May Arsenale
M30 Water Feb Riva Sette Martiri
M216 Ulva Mar Arsenale
M292 Ulva May Riva Sette Martiri
M295 Ulva May Riva Sette Martiri
M169 Water Mar Riva Sette Martiri
M323 Water May Lido laguna
M22 Water Feb Riva Sette Martiri
M34 Water Feb Riva Sette Martiri
M27 Water Feb Riva Sette Martiri
M110 Water Mar Lido laguna
M111 Water Mar Lido laguna
M214 Ulva Mar Arsenale
M228 Undaria Mar Arsenale
M42 Ulva Feb Riva Sette Martiri
M3 Water Feb Riva Sette Martiri
M124 Sargassum Mar Lido laguna
M133 Water Mar Riva Sette Martiri
M113 Water Mar Lido laguna

Supplementary Figure S6. Examples of large macroalgal growth and colonization around the historical centre of Venice. All photographs from this figure were taken by Gian Marco Luna and Grazia Marina Quero.

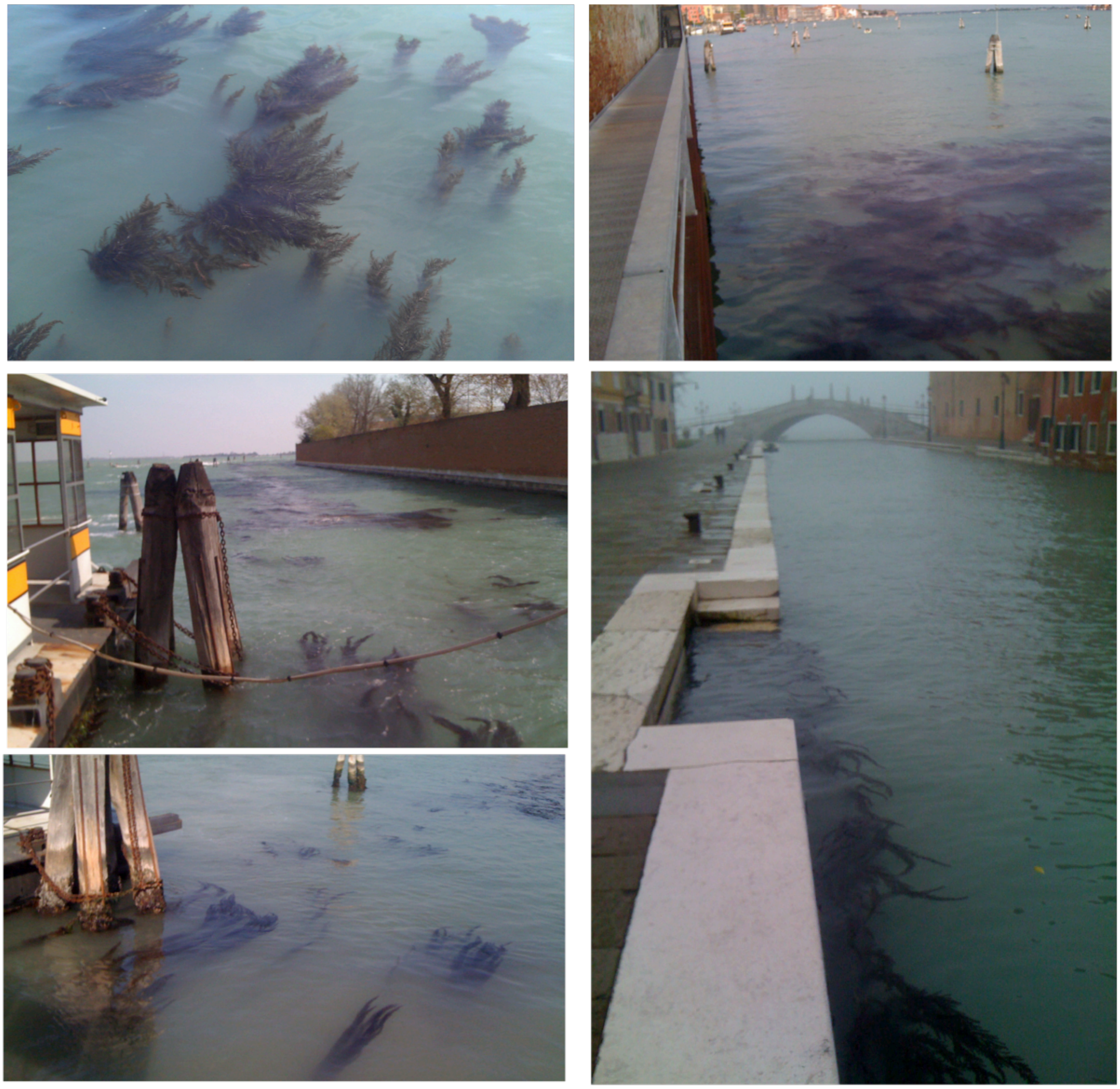

Supplement: Supplementary Information [file srep10969-s1.pdf]
